# Rhino-Orbital-Cerebral Mycosis and Extranodal Natural Killer or/and T-Cell Lymphoma, Nasal Type

**DOI:** 10.3389/fmed.2022.851208

**Published:** 2022-06-17

**Authors:** Dong Ming Li, Li De Lun

**Affiliations:** ^1^Division of Dermatology and Mycological Lab, Peking University Third Hospital, Beijing, China; ^2^Division of Nephrology and Rheumatism, Air Force General Hospital PLA, Beijing, China

**Keywords:** lethal midline granuloma, *mucor irregularis*, *Rhizopus arrhizus*, rhino-orbital-cerebral mycosis, facial destruction, extranodal nK/T-cell lymphoma, nasal type

## Abstract

**Background:**

Extranodal natural killer/T-cell lymphoma, nasal type is a syndrome of middle face destruction with an association to Epstein-Barr virus. Fungi have been recovered from the diseased tissue now and then but were often seen as a lymphoma-associated secondary infection. However, there are ENKTL-NT cases with the recoveries of fungi and complete recovery with antifungal therapy, which are quite similar to rhino-orbital-cerebral mycosis (ROCM) that often confuses the physicians.

**Methods:**

We searched Medline for English-language manuscripts limited to “human” and “case reports,” “letters,” “reviews,” and “clinical conferences” from 1966 to 2022. We used MeSH terms “lymphoma, extranodal nk-t-cell” [MeSH Terms] or “lethal midline granuloma” [MeSH Terms], in combination with MeSH terms “microbiology” [subheading] or “microbiology” [all fields] or “fungi” [all fields] or “fungi” [MeSH Terms] for ENKTL-NT with infections. We used MeSH terms “Mycoses” in combination with “Nose” [Mesh] OR “Orbital Diseases” [Mesh] for rhino-orbital-cerebral fungal infections.

**Results:**

We appraised 149 included articles and extracted references related to ENKTL-NT and/or ROCM. Themes and subcategories were subsequently derived. Our findings revealed that ROCM and ENKTL-NT are characterized by progressive and destructive ulcers in the midline face or rhino-orbital structures. ROCM is mainly caused by fungi in the order of Mucorales, and ENKTL-NT is usually associated with Epstein-Barr virus and sometimes fungi. Radiologically, both are characterized by non-specific features of sinusitis, soft tissue infection, and necrosis. Pathologically, ROCM and ENKTL-NT share the same characteristics of inflammation, necrosis, and granuloma. ROCM is characterized by the detection of fungi in tissue, while ENKTL-NT is typically positive for NK/T-cell markers and cytotoxic granule-associated proteins, proliferation, and vascular damage of angioinvasion, which could be incited by *Mucor irregularis* and *Rhizopus arrhizus* in patients and mice.

**Conclusion:**

ENKTL-NT and ROCM share many similarities in clinical presentations, radiology, and histopathology, and might have the same etiology. This may explain why the two diseases are tangled together in the reported cases, and suggests the role that the fungi may play in the development of these ENKTL-NT/ROCM diseases. The reason why ENKTL-NT and ROCM are sometimes confused is that the main pathogens of ROCM, *Mucor irregularis* and *Rhizopus arrhizus*, are the fungal causative agents of ENKTL-NT.

## Introduction

Extranodal natural killer or/and T-cell lymphoma, nasal type (ENKTL-NT, syn. lethal midline granuloma, LMG) is a clinic-pathological entity characterized by progressive midline necrosis syndrome (MNS) that is predominantly reported in East Asia and parts of Central and South America with high mortality rate. Advanced ENKTL-NT is highly aggressive with survival periods spanning from days to months (1–3). The disease was first described as malignant granuloma associated with the rapid destruction of the face and nose by Mcbride in 1897 (4, 5). Since then, it had been renamed as non-healing granuloma, granuloma gangraenescens, malignant granuloma, idiopathic midline destructive disease, midline granuloma syndrome (MGS), and lethal midline granuloma, all of which are descriptive names with unknown etiological factors (6–9). In histology, the disease is described as inflammatory necrosis, associated with polymorphic cell infiltration and granulation that are mainly Wegener’s type, Stewart’s type, or Tsokos’s type (10, 11). In 1982, Ishii et al. first introduced the term “nasal T-cell lymphoma” when he observed T cells in the lesion, but later he named it as angiocentric T-cell lymphoma with vascular infiltration (12). Since 1999, the disease has been designated as an NK/T-cell lymphoma and is formally termed as “extranodal natural killer NK/T-cell lymphoma, nasal type (ENKTL-NT),” as the pleomorphic cells often express natural killer cells, and less frequently, T-cell markers with atypical cell type (3, 13). Therefore, it was formally incorporated into the classification of hematologic malignancies by World Health Organization (WHO) (3, 14). However, the diagnosis and treatment for ENKTL-NT have been challenging for physicians, as some cases resolved without any therapy (15) or without anticancer therapy and with antifungal therapy only, similar to primary rhino-orbital-cerebral mycosis (ROCM) (16, 17).

Similar to ENKTL-NT, ROCM is also characterized by progressive destruction of the sinus, nose, face, palate, orbit, and other midline structures; proptosis, ptosis, ophthalmoplegia, eyeball necrosis, and vision loss; and pathological features of inflammation, necrosis, and granulation (18, 19). ROCM is featured by the detection of fungi in the tissue, while ENKTL-NT is typically associated with NK/T-cell infiltration, hyperplasia, and angiocentric characteristics. Diagnosis is often confusing when both these features are present in the tissues of patients with facial destruction (16, 20, 21).

In published reports, there were ENKTL-NT patients with fungus recovery who continued to accept anticancer therapy and got worse or even died (2, 22). There were also ENKTL-NT patients who received chemotherapy, died, but turned out to have Mucorales fungal infection in the post-mortem of the nasal cavity (23, 24). In some ROCM patients, the detection of NK cells or/and T cells in tissue led to the change of diagnosis from ROCM to ENKTL-NT and then ended with fatal outcomes (1, 25). A physician reported two ROCM cases with NK/T cells and angiocentric characteristics. Diagnosis for one patient was from ENKTL-NT to ROCM, as fungal mycelium was seen and recovered from tissue, while the diagnosis for the other was from ROCM to NK/T-cell lymphoma because the NK/T cells were seen in the tissue. All these findings suggest an ambiguous understanding of the role of fungi in the development of ENKTL-NT (16, 21).

In the cases that we reported in 2012 and 2021, *Mucor irregularis* and *Rhizopus arrhizus* were identified as the cause of ENKTL-NT, and the patients got complete remission with amphotericin B (AMB) (16). Systemic literature reviewing study showed that *M. irregularis* and *Rhizopus arrhizus* infections in the central face share the same clinic-pathological characteristics with ENKTL-NT. Following our report, some dermatologists presented similar MNS cases, which were confirmed as *M. irregularis-*associated ROCM infections, and all achieved complete remission after receiving AMB (26, 27). With all the above-mentioned clinical evidence and relevant pathohistological and immunohistological information, ENTL-NT and ROCM seem to be tangled in terms of etiology, histopathology, and immunohistology, and the role of fungal infection has not yet been clarified and needs further investigation. We hereby reviewed comprehensive literature in order to explore the etiology, clinical presentation, histopathology, immunohistology, diagnosis, and treatment of the two diseases, the role of fungal infection, and challenges in the diagnosis of ENTL-NT and ROCM.

## Materials and Methods

We searched Medline for English-language manuscripts limited to “human” and “case reports,” “letters,” “reviews,” and “clinical conferences” from 1966 to 2022. We used MeSH terms “lymphoma, extranodal nk-t-cell” [mesh terms] or “lethal midline granuloma”[MeSH Terms], in combination with MeSH term “microbiology” [subheading] or “microbiology” [all fields] or “fungi” [all fields] or “fungi” [mesh terms] for ENKTL-NT with infections. We also used MeSH terms “Mycoses” in combination with “Nose” [Mesh] OR “Orbital Diseases” [Mesh] for rhino-orbital-cerebral fungal infections. The results were combined with “humans” [mesh terms] for cases of human infections.

## Results

We appraised 149 included articles and extracted references related to ENKTL-NT and ROCM. Themes and subcategories were subsequently derived. Our findings revealed that the available literature describes that ENKTL-NT has many similarities to ROCM described in the existing literature, particularly that ROCM is caused by *Mucor irregularis* or *Rhizopus arrhizus*.

### Etiology

The etiological agent of ENKTL-NT has been known to be the Epstein-Barr virus (EBV) since Harabuchi et al. first showed the presence of EBV DNA and EBV nuclear antigen in the lymphoma cells obtained from five patients with ENKTL-NT and suggested that lethal midline granuloma is causally associated with EBV (28), which is regarded to be highly correlated with ENKTL-NT, and positive EBV is one of the diagnostic criteria according to the current understandings (3, 28–33). However, there were EBV-negative cases that had fatal outcomes (34–36). A recent meta-analysis of the association between EBV and ENKTL-NT showed that anti-EBV pre-treatment does not result in any benefit in the prognosis of patients with EBV-positive lymphoma but may bring an adverse effect on the survival outcome (31), while another research says that the results are controversial (37). Moreover, seroepidemiologic studies revealed that the worldwide seroprevalence of EBV infection is as high as 90% (38). These findings support doubts that EBV infection alone may be insufficient for tumor development.

The etiological agents of ROCM are a variety of, mostly fast-growing, fungi belonging to the order Mucorales (18, 19, 39, 40), which gave rise to its original name rhino-orbital-cerebral-mucormycosis (41, 42). As more patients were diagnosed, infections were found to be caused by fungi belonging to the class Zygomycetes (43), including *Rhizopus arrhizus* (*Rhizopus oryzae*) (40, 43, 44), *R. homothallicus* (45), *R. rosporus* var. *oligosporus* (46), *R. microsporus* var. *rhizopodiformis* (46), *Mucor irregularis* (16, 27, 26, 47), *Apophysomyces elegans* (41, 48–50), *A. variabilis* (48), *Cunninghamella bertholletiae* (40, 43, 51), *Lichtheimia ramosa* (*Absidia corymbifera*) (52), *L. hongkongensis* (53), *Syncephalastrum racemosum* (54), *Saksenaea vasiformis* (55–59), and *Cokeromyces recurvatus* (60). The disease was then renamed as rhino-orbital-cerebral zygomycosis, but then infections were identified with fungi outside Zygomycetes, even those in different phyla, such as *Basidiobolus ranarum*, *Conidiobolus coronatus* (61), *C. incongruous* (62, 63), *Aspergillus flavus*, *A. fumigates*, *A. granulosus* (64, 65), *Alternaria infectoria* (19), *Arthrographis kalrae* (66), or *Schizophyllum commune* (67). The disease was then renamed as rhino-orbital-cerebral mycosis. Research in 2014 (19) showed that the pathogenic fungi of ROCM have increased to 37 species in 12 orders, but the list expanded to 47 species in 15 orders with the addition of *Tilletiopsis minor* (68), *Saksenaea erythrospora* (69), *Pleurostomophora richardsiae* (70), *Acremonium*, *Phoma* sp. (71), *Apophysomyces ossiformis* (72), and *Scedosporium apiospermum* (73), *Aspergillus nominae* (74), *Mucor menace* (75), and *Lichtheimia ornate* (76) (Table 1).

**TABLE 1:** Aetiological agents of rhino-orbital-cerebral mycosis.

Phylum or subphylum	Ordinal relationship	*Species name*
Ascomycota	Chaetothyriales	*Exophiala asiatica*
Ascomycota	Chaetothyriales	*Exophiala jeanselmei*
Ascomycota	Dothideales	*Aureobasidium proteae*
Ascomycota	Dothideales	*Scytalidium dimidiatum*
Ascomycota	Calosphaeriales	*Pleurostomophora richardsiae*
Ascomycota	Eurotiales	*Aspergillus granulosus*
Ascomycota	Eurotiales	*Aspergillus flavus*
Ascomycota	Eurotiales	*Aspergillus fumigatus*
Ascomycota	Eurotiales	*Aspergillus niger*
Ascomycota	Eurotiales	*Aspergillus nominae*
Ascomycota	Hypocreales	*Acremonium* spp.
Ascomycota	Hypocreales	*Fusarium solani*
Ascomycota	Microascales	*Pseudallescheria boydii*
Ascomycota	Microascales	*Scedosporium apiospermum*
Ascomycota	Microascales	*Scopulariopsis candida*
Ascomycota	Onygenales	*Arthrographis kalrae*
Ascomycota	Onygenales	*Blastomyces dermatitidis*
Ascomycota	Onygenales	*Histoplasma capsulatum*
Ascomycota	Onygenales	*Paecilomyces liIacinus*
Ascomycota	Onygenales	*Talaromyces marnefJei*
Ascomycota	Sordariales	*Chaetomium globosum*
Ascomycota	Pleosporales	*Alternaria infectoria*
Ascomycota	Pleosporales	*Phoma* spp.
Basidiomycota	Agaricales	*Schizophyllum commune*
Basidiomycota	Tilletiales	*Tilletiopsis minor*
Basidiomycota	Tremellales	*Trichosporon asahii*
Entomophthoromycotina	Basidiobolales	*Basidiobolus ranarum*
Entomophthoromycotina	Entomophthorales	*Conidiobolus coronatus*
Entomophthoromycotina	Entomophthorales	*Conidiobolus incongruous*
Mucoromycotina	Mucorales	*Lichtheimia hongkongensis*
Mucoromycotina	Mucorales	*Lichtheimia ornata*
Mucoromycotina	Mucorales	*Lichtheimia ramosa* (*Absidia corymbifera*)
Mucoromycotina	Mucorales	*Apophysomyces elegans*
Mucoromycotina	Mucorales	*Apophysomyces ossiformis*
Mucoromycotina	Mucorales	*Apophysomyces variabilis*
Mucoromycotina	Mucorales	*Cokeromyces recurvatus*
Mucoromycotina	Mucorales	*Cunninghamella bertholletiae*
Mucoromycotina	Mucorales	*Mucor circinelloides*
Mucoromycotina	Mucorales	*Mucor hiemalis*
Mucoromycotina	Mucorales	*Mucor indicus*
Mucoromycotina	Mucorales	*Mucor irregularis*
Mucoromycotina	Mucorales	*Mucor menace*
Mucoromycotina	Mucorales	*Mucor racemosus*
Mucoromycotina	Mucorales	*Mucor ramosissimus*
Mucoromycotina	Mucorales	*Rhizomucor microsporus*
Mucoromycotina	Mucorales	*Rhizomucor rhizopodiformis*
Mucoromycotina	Mucorales	*Rhizomucor stolonifer*
Mucoromycotina	Mucorales	*Rhizopus arrhizus* (*R. oryzae*)
Mucoromycotina	Mucorales	*Rhizopus azygosporus*
Mucoromycotina	Mucorales	*Saksenaea erythrospora*
Mucoromycotina	Mucorales	*Saksenaea ery vasiformis*
Mucoromycotina	Mucorales	*S yncephalastrum racemosum*

*15 orders, 47 species.*

### Fungi and ENKTL-NT

Interestingly, Mucoralean fungi are frequently seen or isolated in ENKTL-NT cases (2, 16, 21, 22, 77, 78). In some patients, the detection of NK cells or/and T cells in the tissue led to the change of diagnosis from ROCM to ENKTL-NT (1, 25), or from ENKTL-NT to ROCM when fungal elements were observed (21), or ENKTL-NT patients died after chemotherapy due to the detection of Mucorales fungal infection on a post-mortem of the nasal cavity (23).

The first confirmed association of fungal infection in LMG has long been traced back to 1965 when Cowen et al. reported midline granuloma syndrome (MGS, previous name of LMG) in a 22-year-old white male Colorado farmer who turned out to be positive for infection with *Cephalosporium* fungus. The fungus eroded through his hard palate, resulting in destructive changes in the maxilla and mandible, with systemic manifestations of fever, weight loss, and hepatosplenomegaly. Failure of response to surgical drainage, teeth extraction, and treatment with iodides and sulfa necessitated intensive treatment with amphotericin B for 2 months, after which cultures for *Cephalosporium* became negative (79).

In a case that we reported in 2012 (16), *Mucor irregularis* was identified in ENKTL-NT (20). The fungus eroded through his sinuses and hard palate, resulting in destructive changes in mid-face, maxilla, mandible, and partial orbit with systemic manifestations of fever, weight loss, hepatosplenomegaly, and anemia. Failure of response to treatment with itraconazole, terbinafine, and steroids, the patient achieved complete recovery with AMB for 2 months, after which cultures for *M. irregularis* became negative. In the subsequent mural experiments, it was revealed, for the first time, that *M. irregularis* could induce NK/T-cell aggregation and atypical hyperplasia in experimental mice (80).

More recently, we reported an *R. arrhizus* infection in a 35-year-old Chinese male with ENKTL-NT (17). The *R. arrhizus* eroded through his hard palate, sinuses, nose, and face, resulting in destructive changes in the maxilla and mandible, with systemic manifestations of fever, weight loss, and hepatosplenomegaly. Failure of response to teeth extraction, surgical drainage, and treatment with itraconazole, it necessitated intensive treatment with AMB for 2 months. The destructed lesion was completely healed and was successfully transplanted with a thick skin graft from his thigh for the reconstruction of the nose and chin. In subsequent mural experiments, the Mucoralean fungus *R. arrhizus* could induce the expression of NK/T-cell markers (CD3+, CD8+, CD56+, TIA1+, GZMB+, and PRF+), proliferation (KI67+), and angioinvasion, as seen in ENKTL-NT that was suggested as the cause of ENKTL-NT (17, 80).

### Risk Factors

The predominant risk factor for ROCM is uncontrolled diabetes mellitus, particularly for those patients suffering from diabetic ketoacidosis (1, 16, 19, 39, 40, 44, 46, 68, 81–89), followed by leukemia and other hematologic malignancies (39, 40, 54, 71, 82). Other predisposing factors are malignancy, organ transplant, chronic organ failure, steroid use, burns, immune deficiency, AIDS, addiction to drugs (90, 91), and latent COVID-19 infection (92).

Trauma, wounds, surgeries, and other lesions of the midline face, particularly those of the nose or larynx, tooth extractions, local skin lesions, or cleaning the ear canal are all entries that have been reported to facilitate the entry of fungi into the body (86, 88, 93–95). Rhinitis and sinusitis are the common precursors of ENKTL-NT (1, 2, 12, 16, 25, 87, 96, 97) and ROCM (19, 39, 40, 82, 90, 98).

### Image Characteristics

Both in ENKTL-NT and ROCM, signs of sinusitis, such as fluid density, air-fluid level, sinus expansion, and diffuse mucosal thickening, can be seen in the sinuses with radiographs, CT, and MRI. Extensive soft tissue enhancement, medial wall necrosis, palate perforation, and bone erosion can be revealed in some patients (16, 19, 39, 99, 100).

Orbit opacity with enhanced mass in the cavernous sinus (CS) can be seen if orbit involvement develops. Enhancements of the superior rectus, medial rectus, and lateral rectus can be seen in patients with painful ophthalmoplegia (16, 19, 101).

Thrombosis of the cavernous sinus, visible as an enhanced mass or filling defect, is the striking feature in ROCM that can be seen in ENKTL-NT (Supplementary Figure 3) (16, 19). The thromboses may extend into the optic foramen, often with narrowed or enlarged lumen, or aneurysm. Most commonly enhanced mass image and occlusion can be seen within the CS and internal carotid artery that may extend to other brain artery branches (16, 19, 101).

### Clinical Manifestations

Clinically, ENKTL-NT is characterized by midline necrosis syndrome (MNS), that is, progressive necrosis of the face, nose, and upper airways with symptoms of swelling, ulceration, and perforation; destruction of the central face, the nose, and the orbit; and tissue defection (5, 16, 90, 91, 102–105). The disease usually begins as sinusitis, rhinitis, and rhinorrhea with symptoms of nasal congestion, discharge, epistaxis, nasal pain, and occasionally loss of sense (9, 25, 96, 106, 1, 2). As it progresses, it often spreads to adjacent areas with typical signs of MNS (16, 91, 104, 107).

Rhino-orbital-cerebral mycosis (ROCM) is more likely characterized by rhino (facial)-orbital-cerebral mycosis syndrome (ROCMS), which is progressive rhinofacial destruction, palate perforation (sometimes associated with proptosis, ptosis, and ophthalmoplegia), and symptoms of the brain (19, 61, 85, 87, 89, 108). Initial signs include sinusitis, rhinitis with nasal stuffiness, epistaxis, and facial swelling with pain (83). As the infection progresses, it becomes progressively necrotic in the nose, face, and orbit, which are the signs of MNS. All of these are signs of ENKTL-NT and were seen in *M. irregularis-* and *R. arrhizus-*associated ENKTL-NT/ROCM cases (17, 19, 44, 85).

### Pathological Characteristics

Necrosis and pleomorphic inflammatory granulation are the main features of ENKTL-NT and ROCM (10, 16, 18, 19, 109). Besides, ROCM is featured by the detection of fungi in the tissue (16, 19, 69), while ENKTL-NT is typically associated with NK/T cells, hyperplasia, and angiodestruction (3, 110, 13, 14, 33, 111). In some cases, both features were present in patients with MNS, which made the diagnosis rather confused (16, 20–22, 27).

### Necrosis, Inflammation, and Granulation

Necrosis, acute or progressive, focal or diffuse, commonly ischemic with inclusive vasculitis and giant cell infiltration, is the predominant feature of ENKTL-NT that may follow thrombosis or embolism (12, 16, 13, 19, 20, 112, 113). Sometimes, necrosis occurs suddenly like an acute vascular event (3, 9, 16, 114, 115).

Ischemic necrosis with inclusive vasculitis, thrombosis, or embolism is usually seen in ROCM (39, 16, 19, 48, 51, 52, 62, 64, 116–118). Purulent masses can be detected in the cavernous sinus, ophthalmic artery, and other vessels with inflamed or infracted orbital or cerebral tissue or nerves (19). Under a microscope, angioinvasive fungus can be seen with endothelial injury, vessel wall necrosis, or thrombosis (19).

Pleomorphic inflammatory granulation is featured in ENKTL-NT involving predominantly giant cells, lymphocytes, plasma cells, and multinucleated giant cells (16). Sometimes, bacteria, actinomycetes, mycobacteria, and even *Treponema pallidum* may be detected when fungi are the most commonly isolated pathogens (16, 115, 119). Following an infection, inflammatory cell infiltration and granulation are seen in all the patients with ROCM (19). The inflammatory response comprises abundant giant cells, lymphocytes, plasma cells, and multinucleated giant cells, which may be associated with phagocytosed fungal hyphae or spores (19). Occasionally, microabscesses mixed with hyphae could be observed (19, 101, 120).

#### Vascular Damage of Angiocentricity and Angiodestruction

**FIGURE 1:**
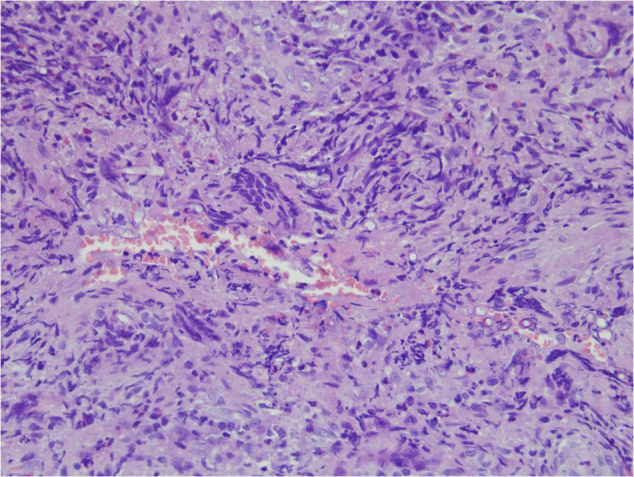
“Extranodal natural killer/T-cell lymphoma, nasal type/rhino-orbital-cerebral mycosis.” *Mucor irregularis* angioinvasion and angiodestruction (H&E, original magnification ×100).

**FIGURE 2:**
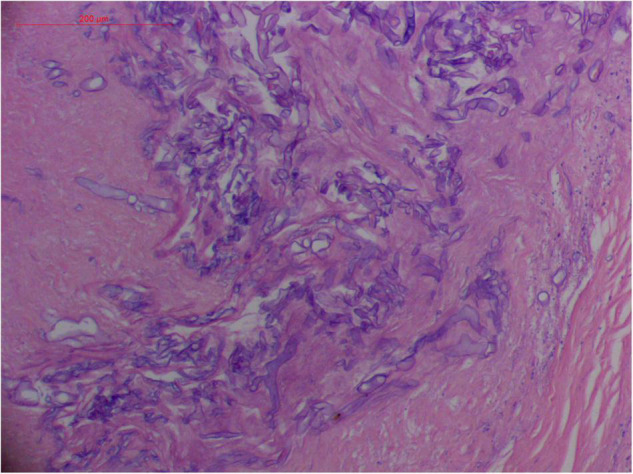
“Extranodal natural killer/T-cell lymphoma, nasal type/rhino-orbital-cerebral mycosis.” Broad thin-walled hyphae of *Rhizopus arrhizus* (H&E, original magnification ×400).

**FIGURE 3:**
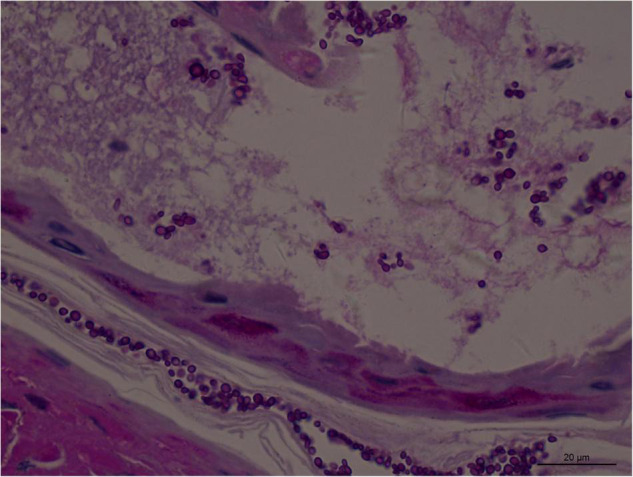
Numerous spores of *Aspergillus sydowii* in the artery lumen and artery wall in rhino-orbital-cerebral mycosis (periodic acid-Schiff staining, original magnification ×1,000).

**FIGURE 4:**
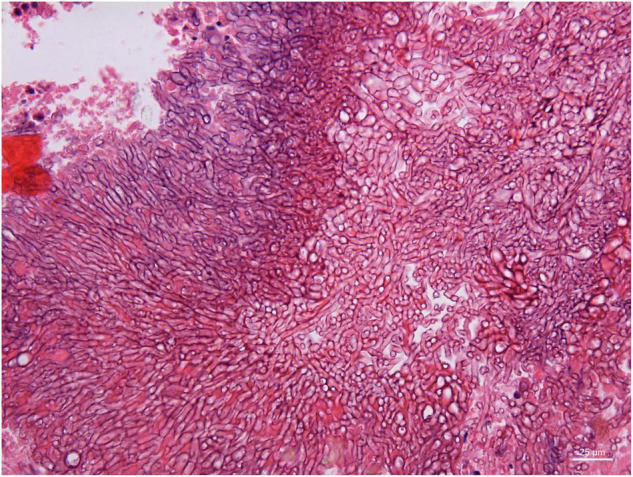
Branched and septate hyphae of *Penicillium* sp. in the cavernous sinus of rhino-orbital-cerebral mycosis (periodic acid-Schiff staining, original magnification ×400).

**FIGURE 5:**
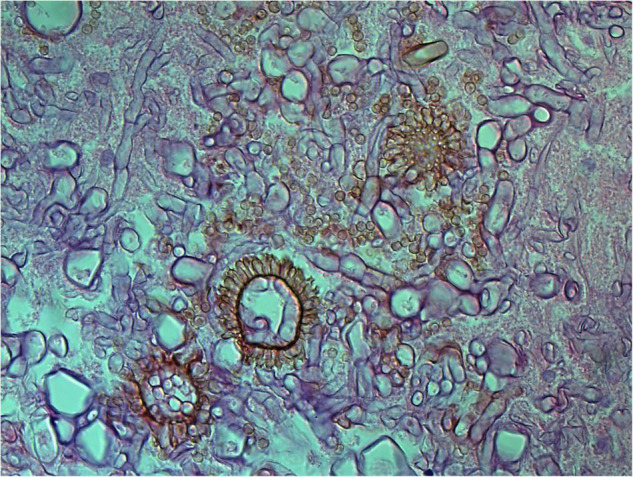
*Aspergillus* heads in the cavernous sinus of rhino-orbital-cerebral mycosis (periodic acid-Schiff staining, original magnification ×400).

**FIGURE 6:**
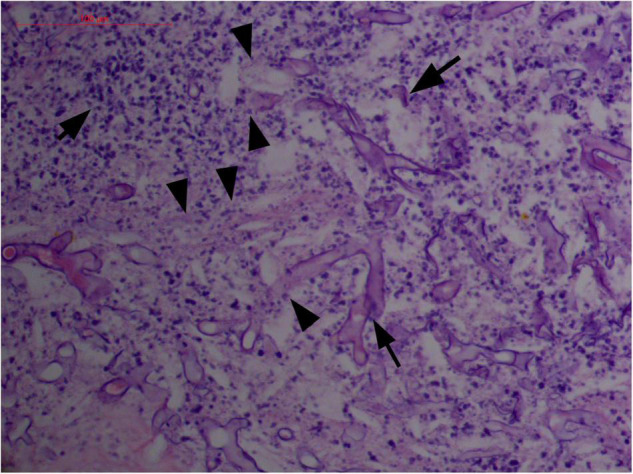
“Extranodal natural killer/T-cell lymphoma, nasal type-orbital-cerebral mycosis.” Distorted, broken, and disintegrated hyphae of *Mucor irregularis* (H&E, original magnification ×400).

Angioinvasive, angiocentric, and angiodestructive infiltration is a well-known pathological feature of ENKTL-NT (13, 3, 12, 16, 18, 20, 106, 112, 113) and ROCM (Figures 1–3) (12, 13, 16, 19, 20, 39, 69, 88, 101, 112, 113). Sometimes, hyphae and spores were seen growing in the artery lumens, on the artery walls, and in the tissues along the destructed arteries (Figures 1–3). Fungal thromboses and artery inclusion were seen in the newly involved tissues, some of which were ischemic necrosis (Figures 5, 6) (16, 19). Meanwhile, fungal elements could be endocytosed and destructed by multinucleate macrophages and lymphoid cells. Angioinvasion, angiocentricity, angiodestruction, and onion-skin lesions could be observed involving destroyed artery, and sometimes hyphae could be seen on the artery wall (Figures 1–4) (9, 16, 121).

#### Polymorphism of Fungi in Tissue

On a hematoxylin-eosin stain, Mucorales would be seen as broad hyphae without septa that could be stained intensely with Periodic acid-Schiff, Grocott’s Methenamine Silver, or Congo red stain, or revealed by fluorescence microscopy after Blankophor staining (26, 39, 69, 85, 46, 16, 40). If branched and septate hyphae are present, the fungus is non-Mucorales, likely an *Aspergillus* sp., *Alternaria* spp., and other non-Mucoralean fungi (Figures 1–6) (19, 120).

#### Atypical Hyperplasia With Ki-67 Expression

**FIGURE 7:**
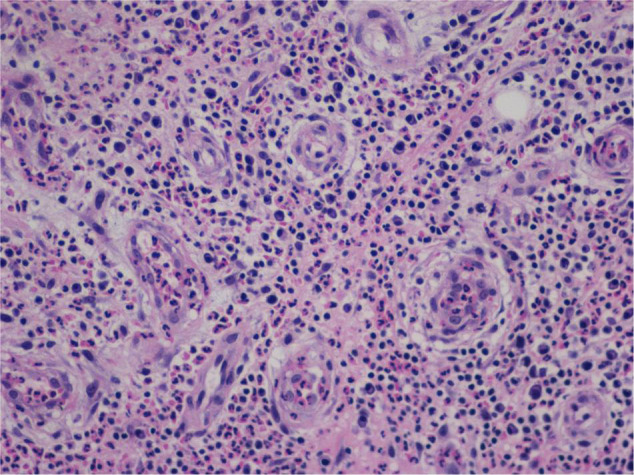
“Extranodal natural killer/T-cell lymphoma, nasal type-orbital-cerebral mycosis.” *Mucor irregularis-*induced atypical hyperplasia (H&E, original magnification ×200).

**FIGURE 8:**
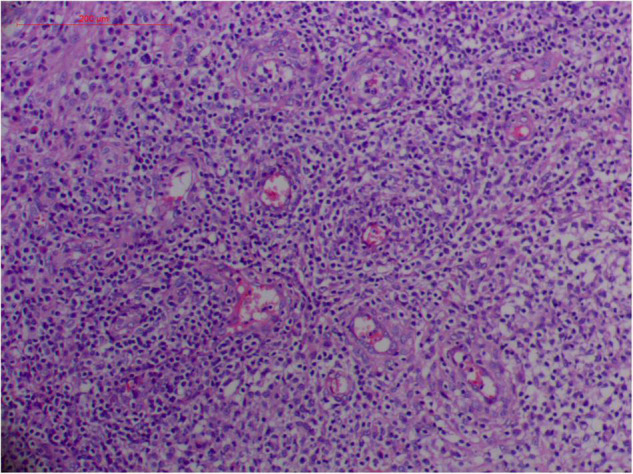
“Extranodal natural killer/T-cell lymphoma, nasal type/rhino-orbital-cerebral mycosis.” *Mucor irregularis-*induced angioinvasion (H&E, original magnification ×200).

**FIGURE 9:**
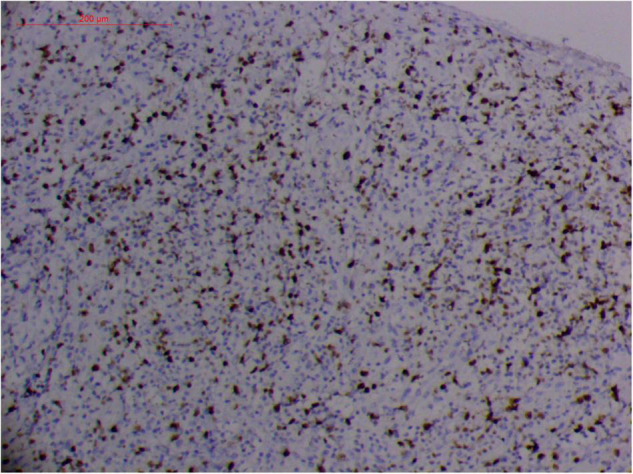
“Extranodal natural killer/T cell lymphoma, nasal type/rhino-orbital-cerebral mycosis.” *Rhizopus arrhizus-*induced positive expression of KI67 from granulomatous tissues (envision method, original magnification ×200).

Granulation with atypical hyperplasia with high Ki-67 expression is usually seen in ENKTL-NT that could either represent neoplastic proliferation (Figures 7–9) (9, 122, 123) or as a marker of activated NK and/or T cells in host defense against pathogens (124). Recently, we reported the inducement of proliferation in ENKTL-NT patients with *M. irregularis* or *R. arrhizus* infection (16) (Figures 7–9). We retrieved, from the peers, a series of reports about the proliferation of cytotoxic T lymphocytes induced by *Staphyloccus aureus* (125), *Nocardia farcinica* (126), *Mycobacterium fortuitum*, *M. marinum* (127), and *Leishmania infantum* (128), all of which were reported to induce ENKTL-NT-like syndrome (129–132). Still, another research reported the recovery of ENKTL-NT following treatment with sulfamethoxazole and levofloxacin when the infections were confirmed in the blood cultures (133).

Fungal-associated atypical hyperplasia with high Ki-67 expression has been reported previously. Terayama et al. reported the inducement of proliferation in the representative forestomach sections by *Candida albicans* in rats (134). In *M. irregularis* or *R. arrhizus* cases, the proliferation was measured using the Ki-67 labeling index which was observed to be as high as 50%, accompanied by high loads of fungal elements (Figure 14). In the mouse model, when the isolated *M. irregularis* or *R. arrhizus* was introduced, high Ki-67 expression was duplicated. A more significant observation is that Ki-67 expression was observed in the cells around the hyphae, indicating that it was probably the fungal infection that led to hyperplasia.

**FIGURE 10:**
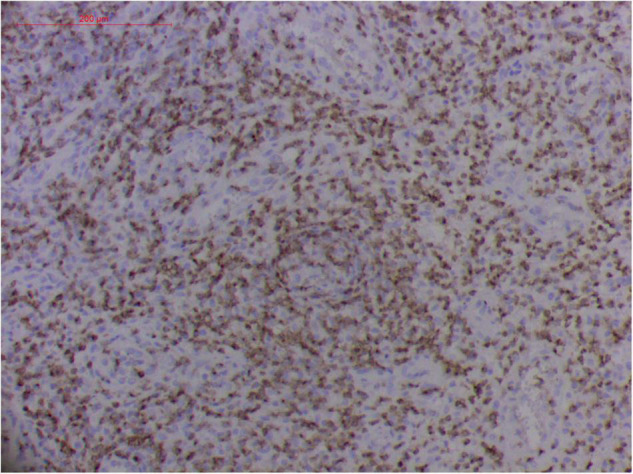
Extranodal natural killer/T cell lymphoma, nasal type/rhino-orbital-cerebral mycosis.” *Rhizopus arrhizus*-induced positive expression of CD2 from granulomatous tissues (envision method, original magnification ×200).

**FIGURE 11:**
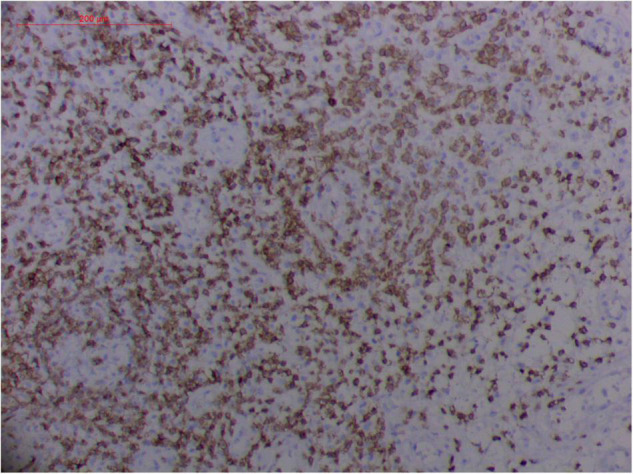
Extranodal natural killer/T cell lymphoma, nasal type/rhino-orbital-cerebral mycosis.” *Rhizopus arrhizus*-induced positive expression of CD3 from granulomatous tissues (envision method, original magnification ×200).

**FIGURE 12:**
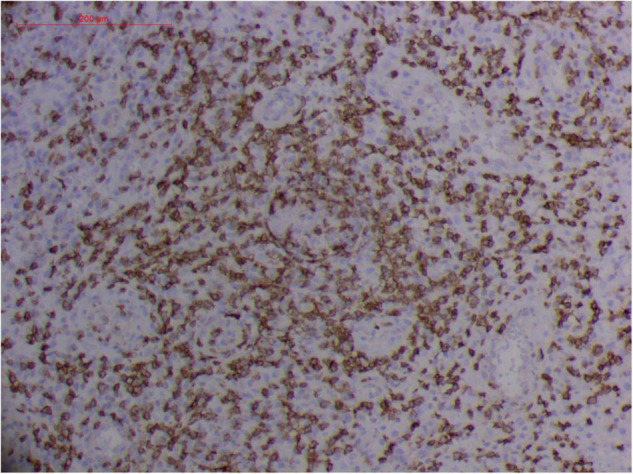
Extranodal natural killer/T cell lymphoma, nasal type-orbital-cerebral mycosis.” *Rhizopus arrhizus*-induced positive expression of CD8 from granulomatous tissues (envision method, original magnification ×200).

**FIGURE 13:**
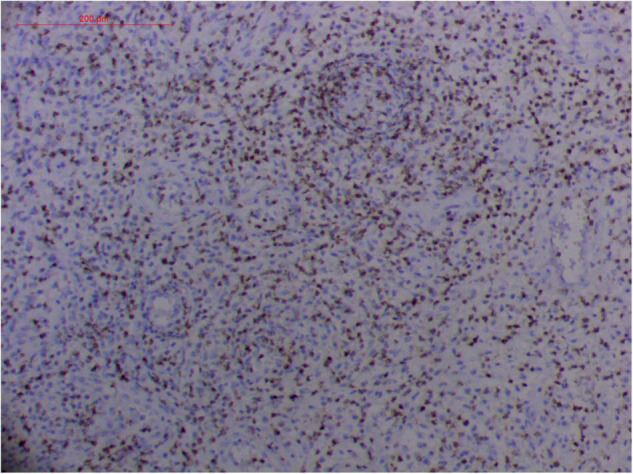
Extranodal natural killer/T cell lymphoma, nasal type-orbital-cerebral mycosis.” *Rhizopus arrhizus*-induced positive expression of GZMB from granulomatous tissues (envision method, original magnification ×200).

**FIGURE 14:**
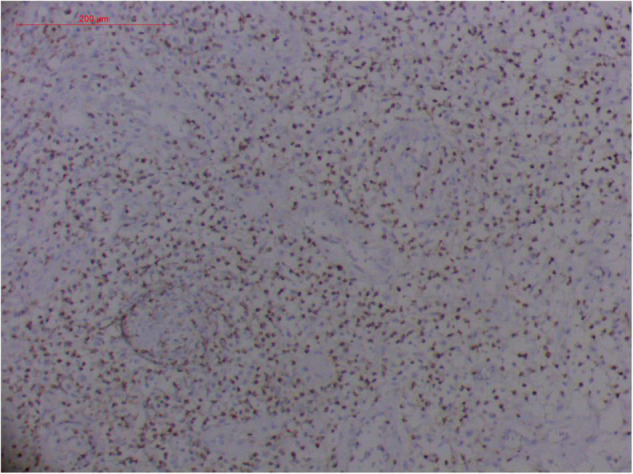
Extranodal natural killer/T cell lymphoma, nasal type/rhino-orbital-cerebral mycosis.” *Rhizopus arrhizus*-induced positive expression of TIA1 from granulomatous tissues (envision method, original magnification ×200).

#### T Cells and/or Natural Killer Cells, and Cytotoxic Granule-Associated Proteins

Pathologic findings of ENKTL-NT show a diffuse infiltration of pleomorphic large and small lymphoma cells mixed with various inflammatory cells on the necrotic background. The lymphoma cells express T-cell markers, such as cytoplasmic CD3 (CD3ε), CD2, and CD8, as well as the NK-cell marker CD56. Cytotoxic granule-associated proteins (GZMB, PRF, and TIA1), Fas ligand, intercellular adhesion molecule-1 (ICAM-1), and perforin are also expressed in the ENKTL-NT cells, which constitute the diagnostic criteria (3, 10, 12, 13, 14, 33, 91, 103, 111, 115).

However, the expression of these T-cell or/and NK-cell markers can be seen in ROCM or ROCM-associated ENKTL-NT, although the roles of these immune cells remain ambiguous (16, 22, 91). In both *M. irregularis* and *R. arrhizus* related ROCM/ENKTL-NT cases, T-cell and NK-cell infiltration were observed (Figures 8–12) (16, 19). Other researchers have reported that in some ENKTL-NT patients, these immunopathological markers were induced by microbes like *Staphyloccus aureus* (131), *Nocardia* sp., *Mycobacterium fortuitum*, *M marinum*, *Pseudomonas aeruginosa*, and *Leishmania* spp. (77, 129–133). All these cases achieved complete resolution after anti-infection therapy. This might be explained by the findings of some previous studies. Potenza et al. observed that Mucorales-specific T cells emerged during the course of infection in patients with invasive mucormycosis and that they exhibited direct antifungal activity comparable to that of either polymorphonuclear leukocytes or antigen-presenting cells (135). Other experiments showed that human NK cells or T cells could damage *R. arrhizus* and other fungi by perforin- and granzyme B-mediated apoptosis and by inducing other immune response cells to bind and then kill fungi (136, 137). Our report showed that the expression of NK/T cells and proliferation were intensified around fungal hyphae, which also suggested that immune response induced cell aggregation and proliferation (16, 17, 80).

### Challenges in the Diagnosis of ENKTL-NT and Rhino-Orbital-Cerebral Mycosis

The clinical-pathological manifestations of ENKTL-NT and ROCM share similarities, including necrotizing lesions; destruction in the upper aerodigestive tract, sinuses, orbits, hard palate, and middle face; inflammation; granulation; and vascular damage (3, 13, 14, 16, 19, 33, 85, 104, 110, 111, 138). The main distinguishing feature between the two is that ROCM is characterized by the detection of fungi in the tissue, while ENKTL-NT is typically associated with NK/T-cell infiltration, with cytotoxic granule-associated proteins of GZMB, PRF, and TIA1 and atypical dysplasia. If the pathology is positive for fungi, the diagnosis is often ROCM; if positive for NK/T cells plus cytotoxic granule-associated proteins, vascular damage, and atypical dysplasia, the diagnosis is confirmed as ENKTL-NT (3, 13, 14, 16, 20, 21, 33, 110, 111).

However, what is challenging is not the established diagnostic criteria but the interesting cases presented by the author and other researchers, where for one reason or another, the decisive evidence, that is, positivity for NK/T cells and positivity for fungi, was found to be coexisting. Whatever diagnosis was made in these cases, anti-infection treatments alone or in combination with anticancer therapy were associated with positive health and life, whereas anticancer treatments alone were associated with fatal outcomes (1, 16, 20, 21, 23, 25, 139). These cases made us ponder the fungal etiology of ENKTL-NT, and further its treatment and prognosis, specifically the relationship between ENKTL-NT and fungal infection.

One major challenge in the dilemma related to the diagnosis of ROCM/ENKTL-NT is the detection and recovery of fungi from the patient specimen (78). As in the cases of *M. irregularis* and *R*. *arrhizus* infections, it was rather difficult to recognize the broad thin-walled hyphae in the granulomatous tissue or in the necrotic areas. By comparing multiple specimens of biopsied samples obtained from the edge of newly infected tissues with those obtained from necrotic tissues that were infected for a long period, we found that typical hyphae could be seen clearly only in the newly infected tissues but were hardly recognized in the necrotic tissues (Figures 4–7, 13) (16, 17, 140). However, they could sometimes be recovered in the culture (16). This observation was in accordance with Wang’s experiment, who observed that *M. irregularis* could not be recognized but could be transferred from the infected tissue to the mouse (140). When *M. irregularis* and *R. arrhizus* were inoculated into the mice, some hyphae were broken down and some were surrounded by giant cells. But more importantly, the inoculated fungi induced the expression of NK/T-cell markers and hyperplasia (CD3+, CD8+, CD56+, TIA1+, GZMB+, and PRF+) in mice, which is considered a diagnostic feature of ENKTL-NT.

As Koch’s four postulates defined, the causal relationship between a microbe and a disease is established when a microorganism can be isolated from the diseased tissue, grown on a pure culture medium, and be introduced into healthy organisms, and subsequently can be re-isolated from the inoculated diseased experimental host (141, 142). In the ENKTL-NT cases, microbes *M. irregularis* and *R. arrhizus* were isolated from the diseased tissue of the ENKTL-NT patients, grown on a pure culture medium, and could be introduced into a healthy mouse, and could be further re-isolated from the inoculated diseased experimental host, indicating the possible causal relationship between the Mucoralean fungi and ENKTL-NT. The causal relationship was further confirmed by the fact that the lesions got completely resolved after treatment with AMB alone.

In the field of oncology, the direct causal relationship between cancer and a microbe was traced back to 1908 when Ellermann and Bang suggested a viral etiology by employing cell-free tumor extracts (143). In 1911, Peyton Rous established an association between cancer and an infectious agent, the Rous sarcoma virus (144). In 1964, Anthony Epstein, Bert Achong, and Yvonne Barr discovered the first human tumor virus, which was later named EBV, from equatorial African pediatric patients with Burkitt’s lymphoma by observing viral particles in the cell cultures (145). Further experiments confirmed EBV as the causative agent of endemic Burkitt’s lymphoma and many neoplasms, including ENKTL (146). In 1990, Harabuchi et al. first showed the presence of EBV DNA and EBV nuclear antigen in the lymphoma cells extracted from five patients with ENKTL-NT and suggested that lethal midline granuloma is causally associated with EBV (28). To date, EBV, Kaposi’s sarcoma-associated herpesvirus, human high-risk papillomaviruses, Merkel cell polyomavirus, hepatitis B virus, hepatitis C virus, human T-cell lymphotropic virus type 1 (HTLV-1), and the Gram-negative bacterium *Helicobacter pylori* have been classified as type 1 carcinogenic agents (147). In recent years, Marseillevirus, *Campylobacter jejuni*, *Chlamydia psittaci*, *Borrelia burgdorferi*, and *Coxiella burnetii* have also been added to the cohort of causal inference between microbes and lymphoma (148). In this study, *M. irregularis* and *R. arrhizus* were demonstrated in the lymphoma cells extracted from ENKTL-NT and experimental mice, and hence should be added to the list of causal microbes of ENKTL-NT, in addition to EBV (16, 17, 80).

### Treatment Options

Treatment options for ENKTL-NT and ROCM are quite different. While ENKTL-NT is considered a malignancy, its treatment is often anticancer therapy, which includes chemotherapy, radiotherapy, radiochemotherapy, and/or stem cell transplantation. In the last few years, chemotherapy (dexamethasone, etoposide, ifosfamide, and carboplatin), concomitant with local radiotherapy (RT-2/3DeVIC), was conducted as a phase I/II trial (JCOG0211) and showed a good clinical outcome. More recent experimental data show good results by employing pembrolizumab, nivolumab, or tofacitinib (149).

Treatment regimens for ROCM include antifungal therapy, reversal of the underlying predisposing risk factors, and surgical debridement. A combination of surgery and potent antifungal therapy was significantly better than antifungal therapy alone (19, 84). Since most of the ROCM cases are caused by Mucoralean fungi, AMB is the common choice of potent antifungal drug (52, 88). In occasional refractory cases, posaconazole or isavuconazole may serve as salvage therapy (44). An antifungal susceptibility test is often performed to decide the choice of drug.

## Conclusion

In conclusion, ENKTL-NT and ROCM share many similarities in clinical presentations, radiology, histopathology, and even immunopathology, and may have the same etiology. This may explain why the two diseases are tangled together in the reported cases, and suggests a role that the fungi may play in the development of these ENKTL-NT/ROCM diseases. The reason why ENKTL-NT and ROCM are sometimes confused is that the main pathogens of ROCM, *Mucor irregularis* and *Rhizopus arrhizus*, are the fungal causative agents of ENKTL-NT. Further research on the role of fungi in ENKTL-NT is needed. Based on the above-mentioned evidence, we strongly suggest screening for fungal infection in patients diagnosed with ENKTL-NT and propose antifungal therapy for ENKTL-NT patients with fungal infections.

## Data Availability Statement

The raw data supporting the conclusions of this article will be made available by the authors, without undue reservation.

## Author Contributions

DL and LL: conceptualization, methodology, data curation, original draft preparation, and illustration. DL: conceptualization, editing, and reviewing. Both authors contributed to the article and approved the submitted version.

## Conflict of Interest

The authors declare that the research was conducted in the absence of any commercial or financial relationships that could be construed as a potential conflict of interest.

## Publisher’s Note

All claims expressed in this article are solely those of the authors and do not necessarily represent those of their affiliated organizations, or those of the publisher, the editors and the reviewers. Any product that may be evaluated in this article, or claim that may be made by its manufacturer, is not guaranteed or endorsed by the publisher.
