# Supplementary material for: Rhino-Orbital-Cerebral Mycosis and Extranodal Natural Killer or/and T-Cell Lymphoma, Nasal Type
Source: Front Med (Lausanne). 2022 Jun 17;9:851208. doi: 10.3389/fmed.2022.851208 (PMC9248758; doi:10.3389/fmed.2022.851208)
Supplement: Supplementary file 1 [file Image_1.pdf]

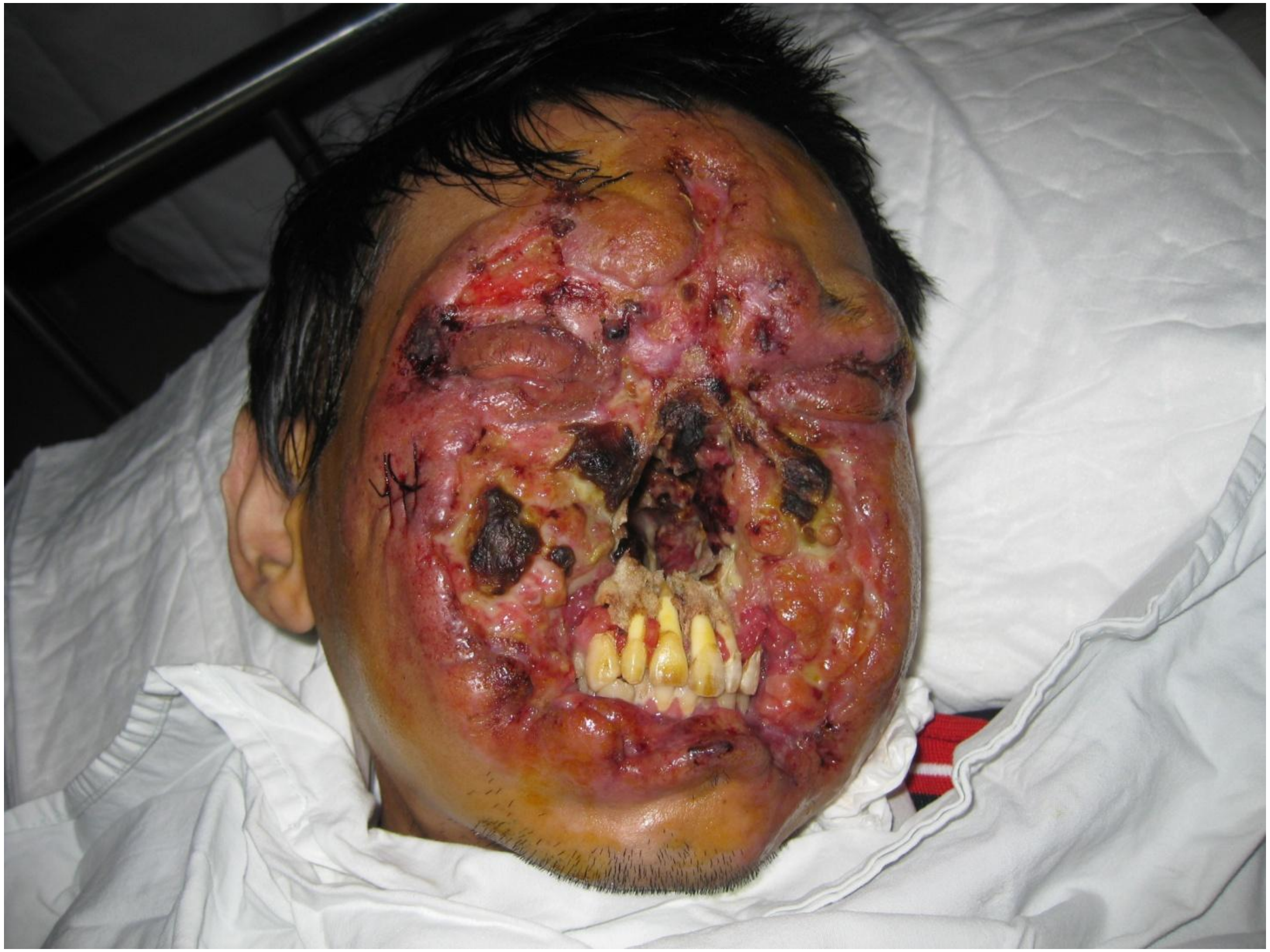

Appendix Figure 1 Extranodal natural killer/T cell lymphoma, nasal type / rhino-orbital cerebral mycosis with *Mucor irregularis* infection: midline necrosis syndrome (MNS).

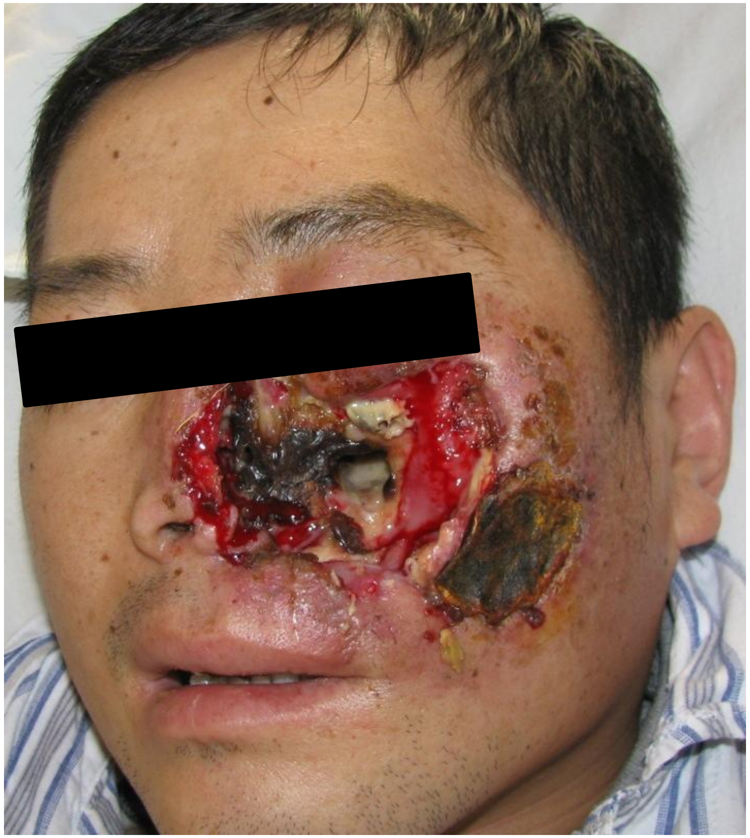

Appendix Figure 2 Extranodal natural killer/T cell lymphoma, nasal type / rhino-orbital cerebral mycosis with *Rhizopus arrhizus* infection: rhinofacial-orbital cerebral mycosis syndrome(ROCMS).

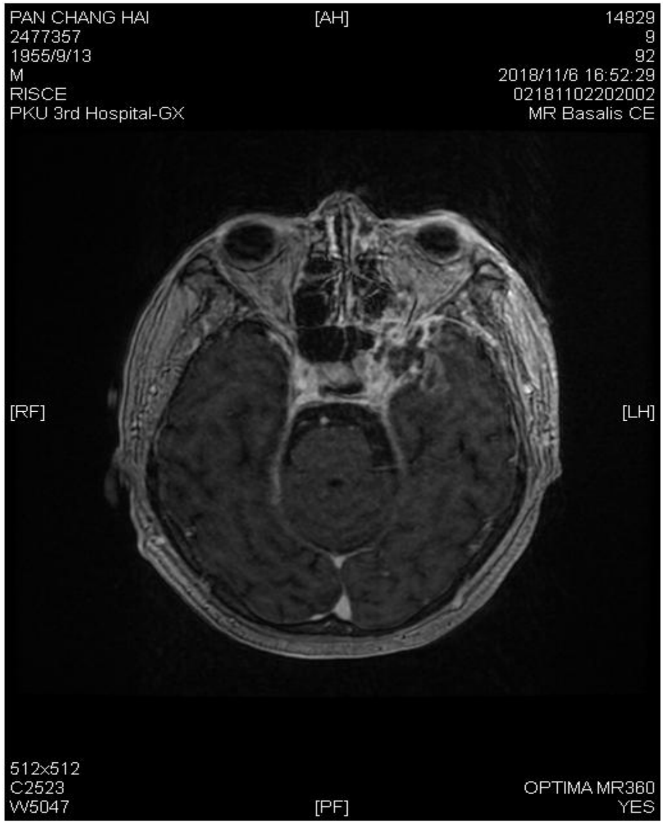

Appendix Figure 3 Rhino-orbital cerebral mycosis with *Aspergillus flavus* infection: (A). MRI demonstrated enhancement of left-sided superior rectus and ethmoidal mucosa thickness.
